# Histoarchitecture of the Ovary of *Rhipicephalus (Boophilus) annulatus* during Pre- and Postengorgement Period

**DOI:** 10.1155/2015/126584

**Published:** 2015-01-12

**Authors:** Sreelekha Kanapadinchareveetil, Leena Chandrasekhar, Jyothimol Gopi, Dibya Ranjan Lenka, Aswathi Vasu, Ajith Kumar KGopalan, Suresh N. Nair, Reghu Ravindran, Sanis Juliet, Srikanta Ghosh

**Affiliations:** ^1^Department of Veterinary Pharmacology and Toxicology, College of Veterinary and Animal Sciences, Pookode, Wayanad, Kerala 673 576, India; ^2^Department of Veterinary Anatomy and Histology, College of Veterinary and Animal Sciences, Pookode, Wayanad, Kerala 673 576, India; ^3^Department of Veterinary Parasitology, College of Veterinary and Animal Sciences, Pookode, Wayanad, Kerala 673 576, India; ^4^Division of Parasitology, Indian Veterinary Research Institute, Izatnagar, Uttar Pradesh 243122, India

## Abstract

The present communication describes the detailed day wise study of histological changes of the ovary of *Rhipicephalus (Boophilus) annulatus* in the postengorgement period together with the systematic classification of their oocytes. The ovary of *R. (B.) annulatus* is panoistic type with an asynchronous development of oocytes. All the stages (II, III, IV, and V) of oocytes except stage I were similar to *R. (B.) microplus*. The stage I oocytes showed basophilia, which was not reported earlier in other species of ticks. Day wise changes were in the form of presence of oogonia in partially fed and day one engorged adults, considerable degeneration of oocytes on day two, emergence of new wave of oocytes on day three, presence of mature oocytes up to day eight, and complete degeneration of ovarian tissue from day eight onwards. The degenerative changes in the ovary appeared initially in the oocytes followed by germinal epithelium.

## 1. Introduction

Ticks are obligate haematophagous ectoparasites of wild and domestic animals including humans. They are the most important vectors of disease causing pathogens in domestic and wild animals [1] and considered second to mosquitoes in their vector potential. It has been estimated that nearly 80 percent of the world's cattle population is exposed to tick infestation [2]. The average tick burden causes an annual weight loss of 0.7 kg/tick [3]. At present, the ticks and tick borne diseases (TTBDs) control is mainly effected by widespread use of acaricides like organophosphates, carbamates, pyrethroids, BHC/cyclodienes, amidines, macrocyclic lactones, and benzylphenyl ureas leading to various problems such as resistance, residues, environmental pollution, and high cost. These factors reinforce the need for alternative approaches to control tick infestations [4]. For the effective tick control around the world, it is necessary to assure the availability of a range of compounds with different modes of action, good safety characteristics for the animals, and soft environmental toxicity profile [5].

The reproductive potential of these acarine parasites is enormous. For the development of newer drugs which target their reproductive system, a thorough understanding of the structure of the ovaries is highly essential. The structure of reproductive system of* Amblyomma cajennense* [6],* Rhipicephalus sanguineus* [7],* Amblyomma triste* [8],* Boophilus microplus* [9], and* Amblyomma braziliense* [10] was documented previously in detail.

*Rhipicephalus (Boophilus) annulatus* is a one-host tick under the subgenus* Boophilus* within the genus* Rhipicephalus* and occurs in greatest abundance in tropical and subtropical regions of the world. It acts as a vector of variety of diseases such as babesiosis and anaplasmosis and also causes a great problem to dairy and beef industry inducing decrease in weight gain and milk production [11–13].* Rhipicephalus (Boophilus) annulatus* is reported as the commonest species in southern region of India [14–16]. Based on the available literature, there is scarcity of information on the structure of ovary of* R. (B.) annulatus*. So the present communication focuses on the detailed study of day wise histological changes in the ovary of* R. (B.) annulatus*.

## 2. Materials and Methods

### 2.1. Ticks

Partially fed (4-5 days prior to complete engorgement) and fully engorged females of* R. (B.) annulatus* were used in the present study. Six partially fed ticks were immediately dissected. Engorged females of* R. (B.) annulatus* (Forty-two specimens) were collected from infested animals and maintained in the biological oxygen demand (BOD) incubator (28 ± 1°C and 85 percent relative humidity). Six fully engorged female ticks were taken out from the BOD incubator on the first, second, third, fifth, and eighth days after engorgement. The ovaries were dissected out in 0.9 percent saline using stereo zoom microscope [17].

### 2.2. Histology

Ovaries were fixed in formaldehyde acetone fixative in the ratio of 9 : 1 [18] for 12 hours at 4°C. Dehydration was carried out in ascending grades of ethanol for 15 minutes each followed by clearing in xylene for 20 minutes. They were embedded in paraffin (melting point 58–60°C). Serial sections were cut at 4 *μ*m thicknesses, stained using hematoxylin and eosin staining method [19], and observed under microscope (Leica, Germany).

The following characteristics were used for classification of the oocytes, namely, size and shape, presence/absence of germ vesicle, cytoplasmic appearance, presence or absence of yolk granules, and presence of chorion [6, 8–10].

## 3. Results and Discussion

Reproductive system functions as the main system necessary for the survival of the tick species. Hence, the detailed study of the morphology and day wise histology of* R. (B.) annulatus* are extremely important and provide newer targets or leads for effective control of these harmful acarine parasites.

**Figure 1:**
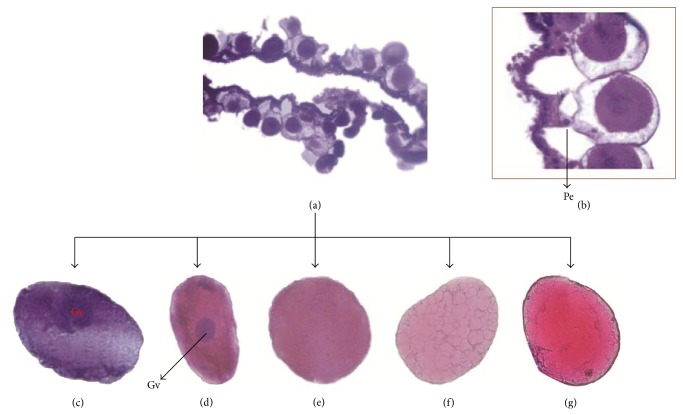
Histological sections of* Rhipicephalus (Boophilus) annulatus* ovary stained by Haematoxylin and Eosin. (a) Ovary with central lumen lined by small epithelial cells interspersed by oocytes in different developmental stages. (b) Pedicel cells attaching the oocytes to the ovary wall. (c) Stage I oocyte. (d) Stage II oocyte. (e) Stage III oocyte. (f) Stage IV oocyte. (g) Stage V oocyte. Pe: Pedicel cell; Gv: Germ vesicle; Y: yolk droplet; Ch: chorion. Bars: (a) 4x, (b) 10x, (c)–(g) 20x.

The ovary of* Rhipicephalus (Boophilus) annulatus* consisted of a horseshoe shaped single continuous tubular structure located at the posterior third of the body. Similar observations were reported in* Dermacentor andersoni, D. variabilis* [20],* Amblyomma cajennense* [6],* A. braziliense* [10], and* Rhipicephalus sanguineus* [7]. The wall of adult tick ovary consisted of central lumen lined by small epithelial cells interspersed by oocytes in different developmental stages (Figure 1(a)). The oocytes were attached to the ovary wall by specialized epithelial cells called pedicel cells [6, 7, 9, 21] (Figure 1(b)) with elongated nuclei. The oocytes were classified into stages varying from I to V (Table 1). Asynchronous development of oocytes in the ovary of* R. (B.) annulatus* was observed in the present study. Similar observations were reported previously [6, 7, 9, 21]. However, synchronous development was also observed in the semiengorged ticks of* A. triste* [8].

In the present study, the oocytes of* R. (B.) annulatus* were classified into five stages similar to* R. (B.) microplus* [9]. However, basophilic nature and saucer shape for the stage I oocytes described in the present study were not reported previously in* R. (B.) microplus* [9]. The basophilia observed could be due to the increased ribosomal content [22]. Stages II, III, IV, and V oocytes of* R. (B.) annulatus* were generally similar to* R. (B.) microplus* [9]. The absence of the germ vesicle in stage III oocytes observed in the present study was not previously reported in* R. (B.) microplus* [9]. Stage IV oocytes of* R. (B.) annulatus* differed from that of* R. (B.) microplus* for the absence of two characters, namely, central large yolk granules arising from smaller granules at the peripheral region and also the presence of germ vesicle. Stage V oocytes of* R. (B.) annulatus* showed heavy deposition of yolk droplets which merged at the center. The fusion of yolk droplets could act as a protective mechanism for cushioning the germ vesicle. Few oocytes were similar to stage VI of* R. (B.) microplus* [9]. However, they were assigned as oocytes with degenerative changes rather than stage VI in the present study.

**Figure 2:**
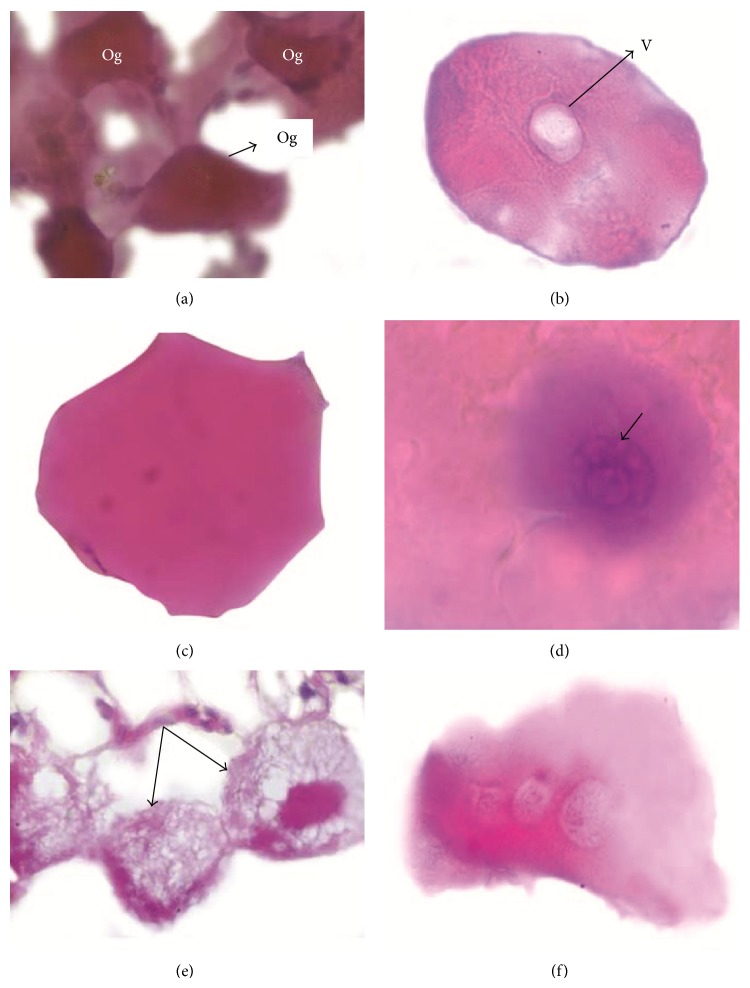
Histological details of day wise changes. (a) Partially fed tick oogonia interconnected among themselves. (b) Vacuole inside oocyte. (c) Polymorphic oocytes. (d) Vacuolations in the nucleolus of germ vesicle. (e) Oocyte with amorphous material. (f) Oocyte fused to form a homogenous mass with series of germ vesicle inside. Og: spindle shaped oogonia; V: vacuole. Bars: (a), (d) 100x, (b), (c), (e), (f) 20x.

Ovary of partially fed* R. (B.) annulatus* tick represented a picture of dense eosinophilic oogonia interconnected among themselves (Figure 2(a)). Oocytes were not differentiated at this stage. Germinal epithelium was observed at a small locus towards one pole. Nurse cells were not seen. However, in the ovary of partially fed* Dermacentor andersoni*, dense eosinophilic oogonia with intercellular bridges were previously described [23, 24]. In panoistic type ovaries, oogonia directly give rise to oocytes without nurse cells [25]. In the present study, oogonia were observed in both partially fed and fully engorged ticks (up to day one after engorgement). However, oogonia were not observed in semiengorged* A. triste* [8]. Observation of the tick's ovary on the day of complete engorgement revealed a similar picture as that of partially fed stage. Oogonia were clearly distinguished as spindle shaped masses having cord-like connections radiating from two or more different sites. The cords gave attachment to large number of small refractile bodies. These refractile bodies could be lipid droplets incorporated into the oocytes after a blood meal. In insects, the digested blood containing a large amount of lipids is secreted to the hemolymph and taken up by the growing oocytes for producing molecules such as vitellogenins [26]. Germinal epithelial cells were more in number in the engorged ticks compared to the partially fed. The nuclear to cytoplasmic ratio of epithelial cells was high. Appearance of dense eosinophilic oogonia and absence of nurse cells strongly suggested that ovary of* R. (B.) annulatus* is of panoistic type.

On day one after engorgement, degenerating oocytes and oogonia were observed along with normal oocytes. Degeneration was observed in few oogonia suggested by the appearance of vacuolations. The first stage oocytes showed strong basophilic reaction indicating active synthesis. Nucleus to cytoplasmic ratio of germinal epithelium was still high as in day zero. The high nuclear to cytoplasmic ratio could be attributed to the active synthetic processes occurring in young germinal epithelial cells. The persistence of oogonia could be due to the new wave of oogenesis occurring in the tick ovary after the degeneration observed at day two.

On day two after engorgement, the ovary of* R. (B.) annulatus* revealed the onset of degenerative changes in oocytes (stages II and III) characterized by vacuolations (Figure 2(b)) which were more pronounced near the germ vesicle. Few oocytes showed polymorphism (Figure 2(c)). Degenerative changes were very distinct towards one pole. These changes were previously reported in ticks treated with extract of* Azadirachta indica* [27] and ricinoleic acid esters from castor oil of* Ricinus communis* [18]. However, in the present study, these changes were seen as a part of the normal development of tick's ovary. The germinal epithelium of* R. (B.) annulatus* showed marked reductions in basophilia. Hence, the multiplication of germinal epithelium occurred during days zero and one of engorgement. Although the oocytes of* R. (B.) annulatus* showed degenerative changes on day two of engorgement, no such changes were observed in the case of germinal epithelium and pedicel cells.

On day three after engorgement, a new population of oocytes was observed, even though degeneration occurred on day two. Eosinophilic reaction was stronger than day two. Eggs were newly formed between forty-eight and seventy-two hours after engorgement in* R. (B.) microplus* [28]. Hence, it was postulated that two waves of oogenesis occur in* R. (B.) annulatus* of which the first one is followed by considerable degeneration, while the second wave persisted during the period of egg laying. Increasing eosinophilia of oocytes observed at this stage was also previously recorded for* R. (B.) annulatus* [29]. On day three after engorgement, oocytes at stage II showed conspicuous cytoplasmic activity. Increase in the number of organelles at the end of stage II and beginning of stage III oocytes was observed for* R. (B.) microplus* [9].

On day four after engorgement, more advanced oocytes were observed at one pole of ovary together with the appearance of few degenerating oocytes, which coincided with the onset of oviposition in* R. (B.) annulatus*.

On day five, vacuolations were observed in the nucleolus of germ vesicle (Figure 2(d)). Degenerative changes and autophagic activity were observed between yolk granules. Most of the oocytes were polymorphic and glassy. The germinal epithelium showed disintegration and loss of cell morphology. The cell boundaries were lost and cells formed a syncytial mass. Stage III oocytes showed vacant spaces and intense trafficking of pinocytic vesicles towards the periphery of the oocytes. Such type of pinocytic activity was previously observed in the stage III oocytes of* R. (B.) microplus* [9]. Autophagic activity was observed in the present study among the yolk droplets. However, autolytic activity was reported previously among the yolk droplets of* R. (B.) microplus* [9]. Vacuolations of the nucleolus of the germ vesicle were also previously described [22]. In the present study, degeneration of oocytes was observed on day two. Degeneration of oocytes and germinal epithelium were observed on day five after engorgement. This observation confirmed the biphasic nature of oogenesis in the ovary of* R. (B.) annulatus*.

On day eight after engorgement, oocytes appeared shattered. A few oocytes showing intense eosinophilia showed basophilic bodies in the cytoplasm. Cytoplasm of all the oocytes showed intense vacuolations and amorphous material (Figure 2(e)). Few oocytes fused and an eosinophilic mass was formed, some of which revealed a series of germ vesicles (Figure 2(f)) and spaces filled with eosinophilic fluid. Nucleus was not clearly distinguished in the germinal epithelium. Basophilia of nuclei of germinal epithelium was markedly reduced along with increase in eosinophilic reaction of its nucleoli. Intense autolytic activity was noticed in some oocytes. Cytoplasmic blebbing over the oocytes suggestive of cell death was noticed. Pedicel cells lost their typical morphology. Nucleus of germinal epithelium showed abnormal morphology and loss of basophilia. Large fluid filled spaces were seen replacing the space occupied by disintegrating nuclei of oocytes. Blebbing and altered nuclear morphology concurred with the description of apoptotic cells [30, 31]. The retention of germ vesicles even when the oocytes fused into a homogenous mass was observed. The germ vesicle remained intact even in adverse conditions [27]. The appearance of amorphous material in the oocytes was observed on day eight. This could be due to resorption which is common in insects due to stress factors such as lack of food, unsuitable environment, conditions of oviposition, and lack of copulation [10]. In* Rhipicephalus sanguineus,* the process of resorption in some oocytes probably occurred as a mechanism to recover certain nutrients [7].

On ninth day after engorgement, the fused mass of germinal epithelium and oocyte formed a syncytium with peripherally arranged nuclei. On the same day, the ovary of* R. (B.) annulatus* presented a picture of complete degeneration.

Hence, it could be concluded that degenerative changes in the ovary of* R. (B.) annulatus* appeared initially in the oocytes and then in the germinal epithelium.

## Figures and Tables

**Table 1:** Classification of oocytes of *R. (B.) annulatus*.

Oocyte I (Figure 1(c))	Oocyte II (Figure 1(d))	Oocyte III (Figure 1(e))	Oocyte IV (Figure 1(f))	Oocyte V (Figure 1(g))
0.03–0.05 mm rounded, elliptical, and saucer shaped	0.06–0.08 mm elliptical	0.106–0.156 mm round	0.217–0.240 mm elliptical to round	0.250–0.380 mm elliptical to round
Germ vesicle present	Germ vesicle present	Germ vesicle not easily discerned	Germ vesicle not visible	Germ vesicle not visible
Homogenous cytoplasm	Homogenous cytoplasm	Fine granulation throughout cytoplasm	Coarse granulation	Coarse granulation
Yolk droplets not formed	Yolk droplets not formed	Yolk droplets not formed	Yolk droplets fully formed	Yolk droplets found to be merging in the centre
Chorion absent	Chorion absent	Thin chorion	Chorion thick	Chorion thick
Dominant on day zero (engorgement day)	Dominant on day zero (engorgement day). Many are destroyed on day two	Dominant on day one (immediately after engorgement). Many are destroyed on day two	Dominant on days four and five (egg laying time)	Dominant on days four and five (egg laying time)
